# Virtual Pathology Education in Medical Schools Worldwide during the COVID-19 Pandemic: Advantages, Challenges Faced, and Perspectives

**DOI:** 10.3390/diagnostics12071578

**Published:** 2022-06-29

**Authors:** Angela Ishak, Mousa M. AlRawashdeh, Maria Meletiou-Mavrotheris, Ilias P. Nikas

**Affiliations:** 1 School of Medicine, European University Cyprus, Nicosia 2404, Cyprus; angela.ishak.10@gmail.com (A.I.); mousa99mahmoud@gmail.com (M.M.A.); 2 Department of Education Sciences, European University Cyprus, Nicosia 2404, Cyprus; m.mavrotheris@euc.ac.cy

**Keywords:** digital pathology, online education, laboratory medicine, histopathology, medical students, distance learning, emergency remote teaching, e-learning, virtual microscopy, anatomy and histology

## Abstract

The COVID-19 pandemic shifted pathology education in medical schools worldwide towards online delivery. To achieve this goal, various innovative platforms were used by pathology educators and medical students, facilitating both synchronous and asynchronous learning. The aim of this study was to review the published evidence regarding remote pathology teaching at the medical school level during this period, present our own experience, and provide some perspectives regarding the best mode of pathology teaching post-pandemic. Among its advantages, virtual pathology education was considered among students and educators as convenient, flexible, and engaging, while learning outcomes were met and students’ academic performance was in general satisfactory. However, several challenges were faced. For instance, suboptimal internet connection compromised the flow of classes and was even associated with a lower academic performance. The lack of hands-on laboratory activities, such as operating the light microscope and tissue grossing, and the reduced student interactions among themselves and their instructors, were also pointed out as significant drawbacks of remote pathology education. Whereas online education has multiple advantages, experiencing the physical university environment, in-person interactions and teamwork, exposure to the “hidden curriculum”, and hands-on activities are vital for medical school education and future student development. In conclusion, the implementation of a blended approach in pathology education—where online and face-to-face sessions are jointly used to promote students’ engagement, interaction with their instructors and peers, and learning—could be the most optimal approach to pathology teaching in medical schools post-pandemic.

## 1. Introduction

Pathology teaching in medical schools worldwide focuses on the study of disease, being the bridge between basic science and clinical practice [1]. Medical students learn the basics of pathology through various teaching modalities including lectures, small group sessions, and assignments as well as traditional and/or virtual microscopy. Recently, there has been a tendency to include more case studies and exercises highlighting clinicopathological correlations into pathology teaching, in order to integrate with other courses within the medical school curriculum and emphasize pathology’s clinical significance in medicine’s multidisciplinary setting [1,2,3]. In general, pathology courses run for a whole semester or more when designed for preclinical medical students [3,4,5,6]. Notably, in some medical schools, senior medical students can additionally choose to attend a more advanced pathology elective which could be 2- to 4- weeks duration. In such electives and in contrast to the preclinical pathology courses, participants are exposed more to pathology as a profession, in addition to the role of pathologists in today’s medical practice, by shadowing residents and attendings, attending grand rounds, and participating in daily sign-out sessions [7,8,9,10,11].

In the pre-pandemic era, most pathology teachings were performed conventionally within the medical schools’ premises [12,13]. However, due to the unprecedented challenges to medical schools worldwide induced by the COVID-19 pandemic, pathology teaching around the world largely shifted towards remote delivery, and whole courses needed to be re-designed to fit the online environment. In addition, pathology educators had to adjust their teaching style, learn novel technologies, and create new material suitable to teach remotely during the unprecedented period they were facing [4,8,11,14,15]. Various innovative platforms were used, designed to facilitate both synchronous and asynchronous learning, as well as small group sessions. Examples of such platforms include Zoom (Zoom Video Communications, Inc., San Jose, CA, USA), Blackboard Learn (Blackboard Inc., Washington, DC, USA), Microsoft Teams (Microsoft Corporation, Albuquerque, NM, USA), Google Classroom (Google LLC., Mountain View, CA, USA), and Google Meet (Google LLC., Mountain View, CA, USA) [8,11,14,16]. Notably, digital pathology was exclusively utilized for the microscopic slide sessions [8,11]. In our pathology sessions and during this period, we used a combination of modalities such as synchronous interactive lectures, laboratory sessions with static images (gross and microscopic) and digital pathology, asynchronous assignments highlighting clinicopathological correlations, and quizzes providing immediate feedback to the students. Furthermore, our exams were administered online using proctoring software [3].

As several pathology educators worldwide have published their experience teaching pathology online to medical students during the pandemic period as well as the reported learning experience of their students, the aims of this review were to:

- Summarize the existing evidence in the literature regarding the advantages and challenges of remote pathology education in medical schools;
- Describe our students’ experience with online pathology education and compare it with what has been reported in the literature;
- Provide some perspectives regarding the best mode of pathology teaching post-pandemic in medical schools.

## 2. Methods

This was a combined study, including both a review of the literature and our own experience regarding remote pathology education—at the medical school level—during the COVID-19 period.

### 2.1. Literature Review

The PubMed database was searched for studies describing changes in pathology education at medical schools worldwide during the pandemic. The following search algorithm was applied: (Pathology OR Histopathology OR Histology) AND medical education AND COVID-19. Initially, the database was searched on 28 January 2022, yet the search was updated on 21 June 2022. Studies describing the pathology education of residents or fellows, pathology practice implications in general, or focusing solely on histology education were outside the scope of this review and were excluded. The article selection was first performed in a title-abstract fashion, followed by a full-text evaluation of articles that fitted the selection criteria. Data extraction was performed by three authors (I.P.N, A.I., and M.M.A.), while any disagreement was resolved by reaching a consensus.

### 2.2. Our Own Experience

To describe our own experience, we mostly used data from our recent survey in addition to a few personal observations from our remote sessions. This anonymous e-survey was delivered to our medical students via Google Forms (Google LLC, Mountain View, CA, USA). The survey was kept open for four weeks, and following its completion, we received and analyzed the answers from 173/255 (68%) of the enrolled students (100 females and 73 males) that participated. Although its quantitative results (including the complete demographic data, predictors of the virtual learning experience, and perceived stress) have recently been published by our team [3], this survey also included some unpublished qualitative data derived from the students’ answers to its five open-ended questions (Table 1) which we aimed to include in the current study and evaluate together with the literature review findings. Analysis of these qualitative data followed a thematic analysis approach [17,18], during which, data were coded and clustered as themes.

Regarding the following chapters of this study, the “Results” section presents the advantages and challenges of virtual pathology education, giving a brief “literature review” coupled with “our own experience” while teaching pathology remotely during the COVID-19 period. “Our own experience” chapters begin by briefly describing the main quantitative findings published in our previous study [3], followed by a summary of the unpublished qualitative data derived from the survey in addition to a few of our personal observations while teaching pathology remotely. The “Discussion” section attempts to interpret the findings presented in the “Results” and provide some perspectives regarding the best mode of pathology teaching post-pandemic in medical schools.

## 3. Results

### 3.1. Literature Search

**Figure 1:**
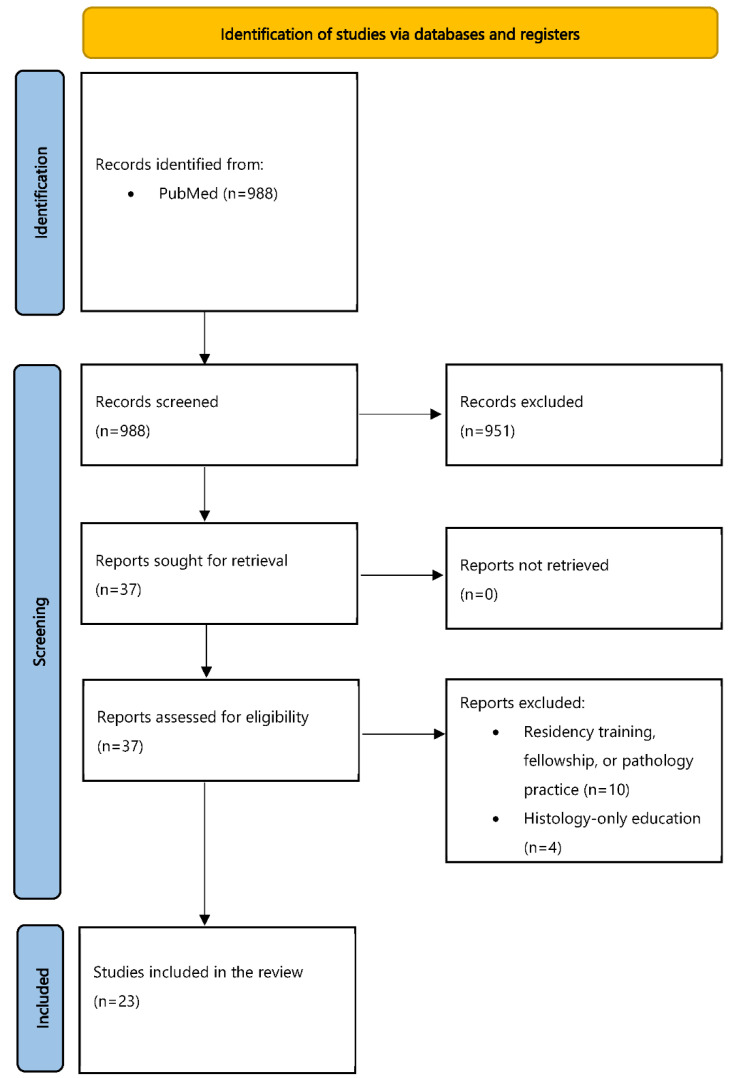
Flowchart of our literature search.

Figure 1 shows the flowchart of our study, following the PRISMA guidelines [19]. A search of the PubMed database revealed 988 articles, the title and abstract of which were screened for eligibility with the objectives of our study. This screening step resulted in 37 articles, and their full text was evaluated by three authors of this study (I.P.N., A.I., and M.M.A.). At this step, 14 articles were additionally excluded; 10 of them described training at the level of residents, fellows, or implications in pathology practice, while 4 focused only on histology education. Subsequently, 23 studies were included in our literature review, and their findings are discussed in the following chapters.

### 3.2. Advantages of Virtual Pathology Education: Literature Review

Our literature review revealed that the remote delivery of pathology courses worldwide was generally followed by highly positive student evaluations, as reported by several authors [3,5,9,10]. Notably, evaluation scores were often improved, compared to the previous years when traditional on-site delivery had taken place [5,9]. Medical students regarded the remote delivery of classes as flexible and less time-consuming, while they appreciated that teaching materials (e.g., session recordings and virtual slides) were available to access anytime and from anywhere [4,10]. On this subject, a group reported creating a YouTube channel containing all the high-quality recordings from their pathology sessions, which was very much appreciated by their students [20]. Furthermore, virtual small group teaching using digital pathology slides was considered a very convenient, engaging, and effective teaching modality by the learners [9]. Similarly, instructors also reported that virtual laboratory sessions were effective in a recent study [21]. Of interest, some students often found the online sessions more interactive, engaging, and better structured compared to the conventional ones, finding it easier to participate and ask questions [4,6]. A study by Rodrigues et al. reported that, although both sexes were asking more questions during virtual classes, female students felt significantly more comfortable doing so than males (48.9 vs. 33%, *p* < 0.03) [4]. Our recent study also showed that the female sex was an independent predictor associated with an enhanced virtual learning experience [3]. Apart from student satisfaction, pathology instructors often addressed that their teaching and even diagnostic skills were improved during the unprecedented period of the COVID-19 pandemic [9]. Furthermore, many of them reported creating new materials and innovative tools to fit their students’ needs while shifting to virtual classes [8]. For instance, Tóth et al. successfully developed 3D autopsy models, using photogrammetry, for their online forensic pathology sessions [22].

In addition to pathology teaching early on in the preclinical medical school curriculum, a number of pathology electives—designed for more senior medical students—were successfully organized around the world during the pandemic period. Parker et al. pointed out the high number of students enrolling in their virtual elective, especially when compared to its conventional equivalent in the previous years (nearly a 10-fold increase); they also addressed the improved attitude of their students towards pathology, as well as their understanding of pathology basics, after they finished this elective [23]. Fu et al. commented on the flexibility of virtual electives to host more students compared to the traditional ones, enhancing their exposure to pathology as a potential future specialization and their understanding of pathology’s crucial role in patient care [8]. In addition, in their survey which was administered pre- and post-rotation, participants claimed they were significantly more likely to choose pathology as their future medical specialty, and reported an improved understanding of what pathologists do or confidence to ask specific questions to them, when their post-rotation survey answers were compared with their pre-rotation answers [8]. Similarly, as shown in another study, students expressed an increased interest in pursuing forensic pathology as their future profession after completing a relevant elective [24]. Of interest, in a study published by Tanaka and Ramachandran [25], their virtual pathology elective received higher evaluation scores than the conventional on-campus elective (4.88 vs. 4.73/5), even from all advanced clinical clerkships (mean = 4.51; range 2.63–5.00), implying that pathology may be a discipline more suitable to virtual learning than other courses. Another elective, organized by the pathology faculty at Weill Cornell Medicine-Qatar, highlighted the virtual elective’s flexibility in place and time, allowing synchronous and asynchronous interactions among students and faculty from multiple institutions and countries, while it was praised by the participants for its high overall quality and versatility [10]. White et al. addressed that their virtual pathology elective was more intensive than their traditional in-person one, and their students got exposed to the same representative cases per rotation regardless of the department’s workload, emphasizing the standardization of teaching, feedback, and student assessment with virtual electives [11]. Lastly, a new website (PathElective; https://www.pathelective.com/, accessed on 7 June 2022) was developed to facilitate pathology e-learning. With PathElective, medical students worldwide can enroll in an organized virtual pathology elective at their own time and pace; multiple modules are included on the website and each contains its objectives, a to-do list, videos, recommended study resources, assignments, and assessments. PathElective received excellent reviews from participants taking this course in a recent survey delivered by its creators [26].

To evaluate a new teaching model, and despite students’ perceptions and virtual experience, a very important parameter is to assess if the learning outcomes are fulfilled and if students’ academic performance is satisfactory. The latter is most commonly assessed by evaluating the examination scores and contrasting them with a gold standard (e.g., examination scores in an already established teaching model). Although the literature is still limited, pathology learning outcomes’ acquisition and academic performance at the medical school level were found to be satisfactory during the pandemic-induced remote course delivery, compared to the gold standard of on-campus teaching. A study by Waugh et al. compared two student cohorts completing the same pathology curriculum, one with remote delivery (during the pandemic) and a prior one taught face-to-face; there was a significant improvement in the mean practical examination overall mark (65.36%  ±  13.12% to 75.83%  ±  14.84%, *p*  <  0.05) for the online student cohort [6]. Likewise, in another study, pathology students attending a virtual pathology course performed significantly better in their practical questions devoid of images (96.5 ± 7.0 vs. 91.2 ± 15.2; *p* = 0.004) and on questions coupled with gross pathology images (88.4 ± 7.5 vs. 84.4 ± 10.3; *p* = 0.007), as compared to students completing the on-campus course the previous year [5]. Lastly, Krasowski et al. reported similar examination scores among three consecutive years of preclinical pathology teaching, the last of which was conducted virtually [27].

### 3.3. Advantages of Virtual Pathology Education: Our Own Experience

The shift to virtual classes was perceived very positively by our medical students who were satisfied by the organization, lecture and laboratory session delivery, the resources provided, and the overall support they received. They also claimed they appreciated the value of histology and/or pathology to understand disease and that the knowledge obtained was crucial for their future profession. Female sex, better performance in the final exam, lower stress levels, and previous degree-holders were all independent predictors associated with an enhanced virtual learning experience [3].

Unpublished results, resulting from our students’ answers to the open-ended questions of the survey delivered to them, revealed that many enjoyed the virtual pathology sessions, as there was “a lot less fuss” and “fewer distractions” compared to the on-campus sessions. This made it easier for the instructor to lecture without being “constantly disrupted by the noises made by fellow students” and for students to concentrate on the lecture without “any disturbances”. Students could “regulate the volume” and thus could listen clearly to everything the instructor was saying. In addition, many of them found the virtual sessions more engaging and interactive than the face-to-face ones. They argued that the various technological tools used (e.g., virtual laboratory sessions, electronic voting, chats, and discussion forums) enhanced their active participation and learning during the online sessions, while they also made it easier to interact with their instructors and with one another. Others stressed the flexibility accompanying virtual learning, noting that attending courses via online platforms was more convenient and less time-consuming, since “there was no need for time wasted traveling back and forth from the university”. This enabled them to better organize their studying schedule (“we were gaining time”).

Similar to the study by Samueli et al. [28], several of our students stressed the value of the available session recordings (the latter were unavailable during our on-campus sessions), a modality that students found to be extremely useful for study purposes. Of interest, our histology students more often appreciated access to the online recordings and their impact on their studying, compared to the pathology ones, exhibiting a stronger need to re-check the materials after their online delivery. In addition, whereas pathology students reported more commonly that virtual learning was convenient, flexible, and time-efficient, histology students more often claimed that they preferred on-campus than online laboratory sessions, emphasizing the importance of microscopy hands-on exercises and the use of glass slides. Lastly, in accordance with the literature previously presented [4], we also personally observed that some of our students were asking more questions (especially using the chat function) during our virtual, compared to the on-campus, sessions the previous years, often making our sessions lengthier and more interactive. A selection of our students’ comments regarding the advantages of virtual education is shown in Table 2.

### 3.4. Challenges Faced during Virtual Pathology Education: Literature Review

A number of studies reported the presence of technical issues hindering the online delivery of pathology classes, such as the suboptimal internet connection. A high internet speed and bandwidth are necessary, especially while examining digital pathology slides [5,9,10,21,29]. Notably, our recent study revealed that a suboptimal internet connection was associated with a worse final examination performance (*p* = 0.04), while the former was also independently associated with enhanced perceived stress levels [3]. Samueli et al. reported that a few of their students reported technical issues while evaluating the digital pathology slides [28]. In some countries, limited access to computers was also addressed [29]. Considering other challenges, students often found it hard to stay attentive during the online classes [6], facing difficulty in separating work from home [4], or experiencing anxiety [21]. Furthermore, others found remote education hampered peer-to-peer teaching and their motivation in general [4,5].

Regarding remote laboratory sessions, and as shown in various studies, a high number of students expressed their preference to return to their on-campus pathology activities the soonest. A common complaint was the lack of conventional microscopy exercises, and many emphasized the importance of learning how to operate the light microscope [2]. Others mentioned their inability to participate in surgical pathology grossing or the laboratory facilities in general, due to the social distancing measures induced by the pandemic [11,25]. Another drawback of virtual education reported was the decrease in interactions and collaborative work among students and instructors during pathology sessions and the teachers’ inability to observe, interact, and provide feedback the same way, compared to what was happening during the on-site delivery [21,25]. Lastly, organizing and conducting virtual laboratory activities and small-group teaching sessions were reported to occupy a significant amount of time for the educators involved [9,11].

### 3.5. Challenges Faced during Virtual Pathology Education: Our Own Experience

In accordance with the literature, the results of our recent study also pointed out that the internet connection quality was also a significant issue for a few of our students while attending the virtual pathology and histology classes. As stated before, a suboptimal internet connection was associated with a worse final examination performance (*p* = 0.04), while the former was found to be an independent predictor of elevated perceived stress [3].

Based on unpublished data resulting from the participant’s answers to the open-ended questions of our survey, a few of our students addressed the concentration issues they faced during the remote sessions. Specifically, they pointed out that “it was tiring to always be sitting in front of the laptop”, and that this had a negative effect on their focus span and their physical wellbeing (e.g., tiredness, headaches, and eye fatigue). They found it “impossible to concentrate on the screen the whole day” and, as a result, they “couldn’t follow the lecture, as much as in the on-campus classes”. In addition, others reported a few technical issues hampering their learning experiences, such as a weak internet connection or sound problems. According to them, these issues were “tiring” and “particularly frustrating”, or even a “really big problem”, since it led them to be frequently “kicked out of sessions” or even their online exams. Some felt that it was more difficult to ask questions during the virtual sessions, while others did not find virtual labs as useful as those conducted on campus. These particular students noted that virtual labs were “uncomfortable and less useful”, stressing that “the microscope part of lab” cannot be replaced virtually. Lastly, a few participants noted that, due to the lack of face-to-face interaction, engagement in online sessions and communication among each other and with their instructors were much more challenging compared to the on-campus sessions.

In addition, our students often listed various sources of concern they experienced during remote learning, which could have a direct impact on their perceived stress levels. Some mentioned anxiety about exams as a major issue. At the beginning of the lockdown, these students had been worried about the format and procedure of the examinations, and whether these would be different from the type of exams previously administered on-campus. The possibility of proctoring issues during the online exams, falsely perceiving a movement (e.g., looking outside the window) as an attempt to cheat, had also been a source of anxiety for some of them. The possibility of technical issues, not only during the exams, but also during the teaching sessions, was a constant source of concern for a few students, while others reported emotional issues stemming from the COVID-19 pandemic itself, such as anxiety about family members’ health and well-being, uncertainty about family members’ jobs, and lack of motivation to study. Lastly, some participants addressed their concerns regarding the impact of the COVID-19 lockdown on their overall academic progress and performance. A selection of our students’ comments, regarding the challenges faced during the remote delivery of our pathology and histology courses during the COVID-19 pandemic, is shown in Table 3.

## 4. Discussion

Both the “literature review” and our “own experience” showed that virtual pathology education during the COVID-19 period exhibited some advantages, yet significant challenges in its implementation as well. A summary of this information is shown in Table 4.

As briefly described in the aforementioned chapters and outlined in Table 2 and Table 4, the pandemic-induced virtual pathology education exhibited many advantages for both students and faculty. In general, online education was considered to be very convenient, flexible, and engaging, allowing lectures, both big and small group/breakout room sessions, and virtual microscopy labs, while supporting both synchronous and asynchronous teaching modalities [3,8,16]. Several free pathology teaching resources exist online which could be directly implemented into virtual education—for example, the PathElective, PathPresenter, Iowa Virtual Slidebox, Leeds Virtual Pathology Library, and Virtual Microscopy Database (VMD)—while educators worldwide have constantly been creating new material, particularly during the pandemic [3,10,26,30,31]. Especially through the chat function, we and others noticed that some students engage and ask more questions in general [4]; this may result in more interactive and lengthier sessions. Notably, virtual pathology education resulted in students’ satisfactory learning outcomes acquisition and overall academic performance, as shown by comparing pathology exam scores before and during the pandemic in a few published studies [5,9]. However, as evidence is still sparse and these findings could be the result of confounding factors (e.g., different exam structure, level of difficulty, and online setting) other than the virtual teaching itself, more studies are needed to reach reliable conclusions on this matter.

Regarding the use of digital pathology in medical school education, various studies have pointed out its advantages over traditional microscopy, including its flexibility, versatility, efficiency, standardization of both laboratory sessions and exams using high-quality slides, and cost-effectiveness. These support a switch to virtual microscopy, at least for the medical school level [11,31,32,33,34]. Of importance, digital pathology seems to enhance student engagement and collaboration among one another [23,32,33]. In addition, multiple authors have stated that the use of virtual over traditional microscopy does not negatively affect the academic performance of students; in fact, it has been shown to boost it in several studies [35,36,37,38,39]. One common disadvantage of using exclusively digital pathology in medical school education is that students do not learn how to operate the conventional light microscope [32]; this was also pointed out by a few of our students. However, the vast majority of medical students will never have to operate optical microscopy after the first years of medical school or as future physicians. For the ones that exhibit special interest, pathology electives or clerkships could be an alternative option. Consequently, educators and students may best focus on the content (acquisition of pathology knowledge), rather than the tool itself, during pathology sessions [32].

Apart from the advantages of remote pathology education, the implementation of the latter was also followed by several challenges for the stakeholders, including technical problems, screen fatigue, and diminished student interactions among themselves and their instructors, besides their limited exposure to the “hidden curriculum” (Table 3 and Table 4). Regarding remote education, others emphasized the lack of hands-on practical sessions (e.g., traditional microscopy exercises, as mentioned before), specific issues regarding online examinations in general, and the reduced engagement of some of them during the virtual classes [40,41]. Notably, both enhanced and reduced engagement was reported within student surveys in different studies as well as our own [4,6,40,41]. We suspect it could be a matter of learning preference among the medical student community, yet cofounding factors (especially during the COVID-19 period) may have also played a significant role. Parker et al. attempted to enhance student engagement during their online classes by encouraging them to turn on their cameras and speak up throughout the sessions or by providing the session handouts only after the end of the sessions [23]. Others prompted the use of interactive polling software such as Mentimeter (Mentimeter AB, Stockholm, Sweden) and Slido (Slido s.r.o., Bratislava, Slovakia), or gaming applications such as Kahoot! (Kahoot! A.S., Oslo, Norway) in their online sessions [24,42]. However, the benefits of participating in traditional on-campus pathology education in this regard may be impossible to reach simply by its remote delivery while experiencing the physical university environment, in-person interactions, and hands-on activities are undoubtedly crucial for medical school education [43,44].

Given the aforementioned evidence, a few authors have proposed the implementation of a blended approach regarding pathology education in medical schools, attempting to incorporate the best practices of both remote and on-campus teaching. This way, the advantages offered by both could be maximized and the challenges minimized [3,10,45]. Notably, a recent meta-analysis within the spectrum of health education supported blended learning which was reported to result in superior learning outcomes [46]. In a blended model, online learning would complement face-to-face learning rather than replace it, enhancing medical students’ learning experience and academic performance [45]. Bryant et al. utilized a blended approach to teach gross pathology during the COVID-19 pandemic, which was positively welcomed by medical students and staff. In their flipped model, short videos were provided before the laboratory sessions to students, familiarizing them with the teaching material. Then, laboratory sessions took place using small student cohorts, where students rotated through different stations to examine the gross specimens, emphasizing active learning [47]. A few authors have recently pointed out the preference of students for blended learning. For instance, Manou et al. stated in their recent study that most pathology students in their institute exhibited a preference for the integration of e-learning into their conventional on-campus teaching post-pandemic [48]. Similarly, when our students were asked about the best delivery mode of the pathology course as soon as the pandemic finishes, the majority preferred a blended rather than an entirely on-campus or online approach [3]. It is clear to us that all the knowledge and experience gained throughout this pandemic period should not be considered just a short-term adaptation and get discarded to go back to the way things were; they can be used post-pandemic, as they have proved to be beneficial for both learners and instructors. For instance, a few of our students noted in our survey that the at-distance offering of the course provided first-hand experience on how high-quality online teaching and learning is feasible in case the need for at-distance instruction arises again or even as a permanent change. Others saw a great opportunity for a more constructive use of technology in pathology education, noting that the technological tools and applications that had been utilized in our online course (e.g., high-quality session recordings, digital pathology, online polls and quizzes, online case studies, discussion forums, and chat) could also be used in the future to enhance students’ participation, communication, collaboration, and learning [3].

In a pathology teaching blended approach, online learning could focus, for example, on delivering the introductory material and digital slide sessions; this could offer flexibility, innovative teaching solutions, and high-quality recordings, boosting students’ academic performance [3,39,49,50]. On the other hand, on-campus sessions could focus on small group teaching, hands-on exercises, and teamwork, in addition to exposure to the “hidden curriculum” (role modeling and professionalism) [27,51]. Selected tools could also be used for on-campus exercises, for example, gross dissection or fine-needle aspiration biopsy simulation models [52,53]. Our opinion is that student assessment also needs to be conducted on-campus rather than online whenever possible. A few studies dealing with medical or non-medical education have shown that, with online examinations, the prevalence of cheating may increase among students [54,55]. Various institutions, therefore, asked their students to sign academic integrity documents before exams and used certain proctoring programs; however, potential cheating attempts may still be very hard to detect, whereas students often report such programs increase their stress levels [6,55,56]. Lastly, as evidence has so far shown the overwhelmingly positive impact of virtual pathology electives worldwide [8,11,23,26], which are typically conducted later on during medical studies, we believe they should keep their current form with potential minor modifications on a case-by-case basis, according to the feedback instructors receive from their students.

## 5. Conclusions

This study summarizes the existing evidence regarding the advantages of remote pathology education in medical schools worldwide during the COVID-19 pandemic, the challenges faced, and opportunities that have arisen for future implementation. As both online and on-campus pathology education have pros and cons, a blended approach could highlight the best practices of both and minimize the challenges in order to offer the best pathology education to the medical student community. Whereas online education is convenient, flexible, and efficient, experiencing the physical university environment, in-person interactions and teamwork, exposure to the “hidden curriculum”, and hands-on activities are vital for medical school education and future student development. The main challenge in the post-COVID medical education era would be to re-design our pathology courses—including the structure, didactic methodology, and resources—and offer a mixture of student-centered activities, maximizing our students’ engagement, interaction with their instructors and peers, and learning. The technologies employed and experience gained from teaching remotely during the COVID-19 period would need to continue being used and shared among educators worldwide, rather than being discarded and simply returning to the pre-pandemic era and the way things were. The application of state-of-the-art technological tools that promote engagement (e.g., simulations, gaming, and interactive polling) would need to be emphasized in modern medical schools, in addition to the implementation of social media to encourage both on-campus and remote interactions whenever possible [24,42,52,53,57,58]. A number of challenges related to online learning, such as the lack of reliable electronic devices or internet connection for some students, could be potentially overcome with the support of Universities themselves, for instance, by ensuring each student with accessibility issues is provided with a suitable computing device and/or by investing in their library services (e.g., physical space with adequate hardware infrastructure and a reliable internet connection and access to variable e-resources). Lastly, pathology educators would also have to transform themselves digitally, become familiar with novel interactive tools, and develop a creative mindset to teach efficiently and effectively in the challenging post-COVID medical school education era.

## Figures and Tables

**Table 1:** Open-ended questions of the survey delivered to our students.

Were there any aspects of virtual learning that you found better than campus-based learning?
Were there any aspects of virtual learning which were impossible or impractical to follow?
What was your biggest concern/worry related to COVID-19 and its impact on your performance in this course?
What particular difficulties emerged in relation to the remote attendance of this course?
What opportunities for improving the teaching and learning of this course have emerged as a result of it being offered at-distance?

**Table 2:** Selection of comments from our students regarding the advantages of virtual education during the COVID-19 pandemic.

**More engaging and interactive** “Participation was easier for all students during online sessions as well as asking questions.” “I could engage more during the lectures and search fast the net.” “Polls made the experience more engaging.” “In the virtual labs although they were not in the microscope the professor explained us and we could see all the things he was describing much clearer.” “…. being able to type any questions in the chat was definitely helpful for students who are more shy or find it difficult to speak in front of the whole ….”
**Fewer distractions/Easier to concentrate** “We could listen more clearly and concentrate to what the professor was saying.…” “It was easier in the sense of having less distractions since friends weren’t there to distract nor was there excess sound from the class.”
**Flexibility, comfort, and improved time management** “Attending from home is very convenient and less time-consuming.” “Less time consuming overall as we were able to divide the time to study/focus for each class according to our personal needs.” “The freedom of not having to be formal when attending class was nice, meaning we could be in pyjamas and no one would have a clue.”
**Access to high-quality recordings** “Being able to go back to previous lecture recordings was one of the biggest pros with online classes.” “While studying for exams, it was EXTREMELY useful to have access to the recordings.”
**First-hand experience that high-quality online teaching is feasible** “You showed that you can do it remotely, keep it and don’t go back to stone age!!”
**Adaptability skills** “Learned to be flexible in respect of a pandemic.”

**Table 3:** Selection of comments from our students regarding the challenges faced while attending the virtual classes during the COVID-19 pandemic.

**Technical issues** “Sometimes, it was particularly frustrating when my internet would disconnect in the middle of a session.” “And of course, any technical issues during important classes….” “The stress during the examinations because of possible technical problems.” “That based on my poor internet connection, my online exams would crash.”
**Laboratory sessions and lack of hands-on training** “The lab sessions weren’t as helpful as they were on campus.” “I think the microscope part of lab is really important and I think it can’t be replaced.”
**Difficulties in asking questions during the online sessions** “I felt it was difficult to ask questions during the online labs and lectures.” “.... it’s much easier in real life since I can easily raise my hand and just ask or quickly go find you upstairs in your office/after lab.”
**Screen fatigue and concentration issues** “Focusing for a long period of time in front of a screen was difficult.” “It was impossible being in front of a screen literally all day; my eyes were hurting, I constantly had headache, I couldn’t study on my computer more hours etc.” “It’s just too comfortable at home and I need the university area to concentrate properly.” “Long hours in front of a laptop made it unbearable to focus after some point.” “I lived in a place full of family so sometimes it was hard to concentrate.”
**Missing interaction with instructors and peers** “The lack of personal interaction makes it really hard to focus and listen to classes.” “Not having interaction and discussions with my classmates and professors.” “The absence of a face-to-face, more “human” relationship with my instructors.”

**Table 4:** A summary of advantages, challenges faced, and perspectives regarding virtual pathology education, as shown in the “literature review” and “our own experience”.

**Advantages:** Flexibility and improved time management Sessions/teaching materials are available anywhere and anytime Interactivity, more questions asked by some students High-quality recordings Use of innovative teaching platforms New teaching materials and technologies Instructors improving their teaching skills Enhanced enrolment rates in pathology electives Improved attitude towards pathology Enhanced consideration of pathology as a future medical specialty Satisfactory academic performance
**Challenges Faced:** Technical issues Screen fatigue Reduced engagement by some students during classes Instructors’ difficulty in appraising students’ engagement Hard to separate work from home No light microscopy exercises and/or grossing during laboratory sessions Reduced student interactions with instructors and peers Reduced student exposure to the “hidden curriculum” (e.g., role modeling and professionalism)
**Perspectives:** Shift towards a blended approach

## Data Availability

Data are contained within the article.
